# The Sinonasal Outcome Test–22 or European Position Paper: Which Is More Indicative of Imaging Results?

**DOI:** 10.1177/0194599820953834

**Published:** 2020-09-01

**Authors:** Allen S. Zhou, Anthony A. Prince, Alice Z. Maxfield, C. Eduardo Corrales, Jennifer J. Shin

**Affiliations:** 1 Department of Otolaryngology–Head and Neck Surgery, Harvard Medical School, Boston, Massachusetts, USA

**Keywords:** rhinosinusitis, patient-reported outcome measure, position statement, computed tomography, diagnostic assessment, predictive value, telemedicine, COVID-19

## Abstract

**Objective:**

The 22-item Sinonasal Outcome Test (SNOT-22) is a trusted measure of symptom severity in chronic rhinosinusitis. The European Position Paper on Rhinosinusitis (EPOS) provides widely accepted diagnostic criteria, which include sinonasal symptoms, their duration, and imaging results. Our objective was to compare these approaches to assessing symptoms to determine if either was more indicative of radiologic findings, to support decisions in telehealth.

**Study Design:**

Observational outcomes study.

**Setting:**

Tertiary care center.

**Methods:**

In total, 162 consecutive patients provided a structured sinonasal history, completed the SNOT-22, and underwent sinus computed tomography (CT) within 1 month. SNOT-22 scores, EPOS-defined symptom sets, and Lund-Mackay results were assessed. To facilitate direct comparisons, we performed stepwise evaluations of sinonasal symptoms alone and combined with duration. The discriminatory capacity for imaging results was determined through areas under the receiver operating characteristic curves (ROC-AUC) for dichotomous outcomes and ordinal regression for multilevel outcomes.

**Results:**

In ROC-AUC analyses, SNOT-22 and EPOS-defined symptoms had similar discriminatory capacity for Lund-Mackay scores, regardless of duration. Within ordinal regression analyses, SNOT-22 nasal scores were significantly associated with Lund-Mackay scores, while EPOS-defined nasal symptoms were not statistically significantly related.

**Conclusions:**

SNOT-22 nasal scores and EPOS-defined nasal symptoms may have similar associations with imaging results when assessed via ROC-AUC, while SNOT-22 may have more association within ordinal data. Understanding the implications of discrete patterns of symptoms may confer benefit, particularly when in-person and fiberoptic exams are limited.

Chronic rhinosinusitis is a highly prevalent medical condition, affecting 11.6% of American adults.^1^ This condition underlies more than 4.1 million visits to physicians^1^ and an estimated $60 billion in health care expenditures each year.^2^ Visits for affected patients each begin with a staple of the clinical encounter: an inquiry into individuals’ symptoms and relevant history. Prior studies of patient-reported symptoms and diagnostic impressions related to chronic rhinosinusitis have had mixed results, with some data suggesting useful specificity and sensitivity, while others suggest poor discrimination across reports.^3-10^

Chronic rhinosinusitis symptoms have been conceptualized in multiple ways in clinical practice, whether through proposed diagnostic criteria or research tools. For example, the 22-item Sinonasal Outcome Test (SNOT-22) is a validated measure of patient symptom severity increasingly used in routine practice. While originally developed for research, this instrument has demonstrated properties that provide information for clinical decision making and monitoring treatment response.^11-17^ Nasal-specific items within SNOT-22 comprise their own domain, based on mathematical analyses and understanding of typical patient presentations.^7,18^ At the same time, the European Position Paper on Rhinosinusitis and Nasal Polyps (EPOS) has provided widely used diagnostic criteria for chronic rhinosinusitis, which incorporates a clearly defined means of cataloguing specific aspects of sinonasal symptomatology.^19,20^

Particularly in the current clime of telehealth and cost considerations, understanding how specific patterns and details of symptom assessments can predict radiological imaging findings provides important information to support clinical decision making. Previous studies of the relationship between SNOT-22 *overall* score and imaging scores have documented no relationship,^8,21-26^ although *nasal domain* scores may have correlation with computed tomography (CT) findings.^7-10^ Data assessing EPOS-defined symptoms related to imaging findings have been more limited.^27-29^ To our knowledge, the predictive ability of EPOS-defined nasal categorization has not been previously studied in comparison to SNOT-22 assessments. Given the current practice environment, it becomes of interest to assess how we can optimally assess patients’ symptom descriptions to best discern those who would have objective imaging findings. Our objective was therefore to determine whether SNOT-22 nasal domain scores or EPOS-defined symptom sets were more indicative of imaging results. The null hypothesis was that SNOT-22 nasal scores and EPOS-defined symptoms would have no difference in their associations with Lund-Mackay CT scores.

## Materials and Methods

### Study Design and Participants

Approval for this study was obtained from the Partners Healthcare Institutional Review Board before data collection began. Consecutive patients age 18 years or older who presented with a chief complaint of rhinosinusitis or nasal drainage to a participating hospital ambulatory otolaryngology clinic were eligible. Patients were included if they prospectively provided a structured sinonasal history, completed the entire SNOT-22 questionnaire, and underwent sinus CT imaging within 1 month. The structured history included an assessment of symptom duration. Exclusion criteria were the presence of nonnasal chief complaints or lack of either SNOT-22 completion or related imaging within the specified timeframe. Since studies of discriminatory ability ideally include patients with a range of symptom and disease states, symptom thresholds and endoscopy were not a requirement for entry.

### Chronic Rhinosinusitis Symptom Assessments

#### SNOT-22

SNOT-22 is a 22-question validated, disease-specific instrument, which quantifies patient-reported symptoms and functional impact of chronic rhinosinusitis. Assessing both sinonasal and related health,^30^ it has gained widespread use because of its clinical applicability, test-retest reliability, internal consistency, validity, and responsiveness to change.^30-32^ Within the instrument, each symptom is scored from 0 (no problem) to 5 (as bad as it can be). The total overall possible score ranges from 0 (best) to 110 (worst). SNOT-22 focuses on the preceding 4 weeks and contains specific domains. Domain scores are calculated by averaging the scores of questions corresponding to that domain.^18^ A 4-domain system based on mathematical factor analysis and clinical intuition has been described in prior literature.^7^ Here, the nasal symptom domain includes nasal blockage/congestion, altered sense of smell/taste, rhinorrhea, postnasal discharge, thick nasal discharge, facial pain/pressure, nose blowing, sneezing, and cough. Although other domain systems exist,^18,33^ this system was selected because it aligns with traditional clinical diagnostic concepts and has undergone psychometric assessment.^7^

#### EPOS

The EPOS provides vetted guidance for the management of rhinosinusitis and helpfully includes a definition of chronic rhinosinusitis.^19^ Within this definition, diagnostic criteria require 2 or more sinonasal symptoms, one of which must be either nasal blockage/obstruction/congestion or nasal discharge; there should also be concurrent facial pain/pressure and/or reduction or loss of smell. In addition, EPOS diagnostic criteria require a symptom duration of at least 12 weeks and evidence of demonstrable disease on nasal endoscopy or CT imaging.

#### Differences between SNOT-22 nasal domain and EPOS-defined symptoms

While there are similarities in these 2 approaches, EPOS differs from SNOT-22 in multiple ways. More specifically, EPOS focuses on the need for symptom pairs clustered in the pattern described above to be supportive of the diagnosis. EPOS does not include assessments of nose blowing, sneezing, or cough, which are included in the SNOT-22 nasal domain used. Moreover, EPOS focuses on a duration of 12 weeks while SNOT-22 has traditionally queried the patient experience during the prior 4 weeks.

### Radiographic Analysis

CT results were scored using the Lund-Mackay scale due to its widespread, accepted use in chronic rhinosinusitis.^34^ This scale is often favored clinically due to its high intra- and interobserver agreement and responsiveness.^35,36^ In this scale, each paired paranasal sinus and osteomeatal complex is scored from 0 to 2. A score of 0 indicates no abnormality, 1 indicates partial opacification, and 2 indicates complete opacification. An overall score ranging from 0 (no abnormality) to 24 (all areas completely opacified) is then calculated.

### Data Analysis

The primary outcome focused on whether either the SNOT-22 nasal domain or EPOS nasal symptom set was more indicative of radiologic findings of chronic rhinosinusitis. This relationship was assessed via receiver operating characteristic area under the curve (ROC-AUC) analyses for dichotomous outcomes and ordinal regression for multilevel, ordered outcomes. Since ROC-AUC analyses are predicated on normal/abnormal outcome data, we used a Lund-Mackay threshold of either ≥5 or ≥1 to encompass any possible chronic rhinosinusitis finding on CT. A threshold of Lund-Mackay ≥5 was used as some authors have suggested using this cutoff to exclude incidental CT findings nonsuggestive of chronic rhinosinusitis and select for patients who have true chronic rhinosinusitis.^37^ While the rationale here is clear, this approach may also classify as “normal” some true findings of chronic rhinosinusitis that are concentrated or severe in only 1 or a few sinuses; for example, complete opacification of only the bilateral maxillary sinuses would result in a Lund-Mackay score of 4 and be considered “normal” using such a threshold but could be quite clinically significant. In a related but separate analysis, we also evaluated the Lund-Mackay score as an ordinal outcome variable, assessing for a potential relationship between SNOT-22 nasal domain or EPOS symptom results through ordinal regression, which supports analysis of multilevel results.

To facilitate the most direct comparisons possible, we performed stepwise evaluations of sinonasal symptoms alone, then symptoms combined with their duration. More specifically, ROC-AUCs were used to determine the discriminatory ability of SNOT-22 nasal domain and EPOS-defined symptom criteria for Lund-Mackay results, with and without the factor of symptom duration of at least 3 months. ROC-AUC values <0.5 suggested no discriminatory capability, 0.5 to <0.6 low capability, 0.6 to <0.7 mild capability, 0.7 to <0.9 moderate capability, and 0.9 to 1.0 high capability.^38^ Comparisons of the ROC-AUC among the differing approaches to cataloguing sinonasal symptoms with and without duration were assessed to test the null hypothesis that there was no statistically significant difference between these different approaches.^39^ A threshold value of 2 for the SNOT-22 nasal domain score was used in these analyses as it represents mild or worse symptoms and provides a straightforward threshold for clinicians who seek to apply these data in real time.^7,14,40-42^ Comparisons between ROC-AUC values were assessed using established nonparametric methods to test the null hypothesis that there was no statistically significant difference between groups.^39^ The minimum sample size (n = 86) to support a study with 80% power to determine a 0.15 difference between ROC-AUC results within subjects was determined prior to proceeding with the analysis. Ordinal regression was employed according to previously established techniques^43^; within these analyses, the Lund-Mackay score was the ordinal outcome variable, while the SNOT-22 nasal domain and EPOS-defined symptom sets were predictor variables. As multiple comparisons were performed across all of these assessments, we also applied established methods to adjust *P* value significance,^44^ which resulted in a threshold of *P* = .011 to achieve statistical significance.

## Results

### Baseline Characteristics

A total of 162 consecutive patients from participating clinics met criteria for inclusion in the study. The mean age was 46.5 years (range, 18-80), and 68.5% were female (**Table 1**). The median overall SNOT-22 score was 37 (range, 4-102), and the median Lund-Mackay score was 4 (range, 0-17). Within the study cohort, 57.2% applied nasal saline spray and 56.0% used nasal saline rinses either with or without added medications (eg, nasal budesonide). Intranasal steroids were used in 67.2% of patients, nasal antihistamines in 13.2%, and nasal decongestants in 34.0%. In addition, usage of oral steroids, oral antihistamines, oral decongestants, and oral antibiotics was 35.5%, 64.1%, 50.9%, and 67.6%, respectively.

**Table 1.** Patient Characteristics (n = 162).

Demographics	Value
Age, mean (range), y	46.5 (18-80)
Sex, % female (95% CI)	68.5 (60.9-75.3)
SNOT-22 score, median (range)	
Overall score	37 (4-102)
Domain scores	
Nasal	2 (0-5)
Ear	1 (0-5)
Sleep	2 (0-5)
Psychological	1 (0-4)
EPOS-defined symptoms, % positive (95% CI)	88.9 (83.0-92.9)
Lund-Mackay overall scores, median (range)	4 (0-17)

Abbreviations: EPOS, European Position Paper on Rhinosinusitis and Nasal Polyps; SNOT-22, 22-item Sinonasal Outcome Test.

### Symptoms Alone

ROC-AUC analyses were used to assess the discriminatory capability of SNOT-22 and EPOS for Lund-Mackay scores, before the stepwise addition of symptom duration. When sinonasal symptoms alone were considered, relative to Lund-Mackay scores ≥5, the SNOT-22 nasal domain and EPOS nasal symptoms alone showed low discriminatory capability (SNOT-22 ROC-AUC, 0.59; EPOS ROC-AUC, 0.53; *P* = .193; **Table 2**). There was no significant difference between the 2 approaches when assessing sinonasal symptoms alone (**Table 2**). The SNOT-22 nasal domain and EPOS symptom set both also had low discriminatory capability for Lund-Mackay scores ≥1 (SNOT-22 ROC-AUC, 0.59; EPOS ROC-AUC, 0.58; *P* = .886; **Table 2**).

**Table 2.** Receiver Operator Characteristic Areas Under the Curve for SNOT-22 Nasal Domain Scores and EPOS-Defined Nasal Symptoms.

Characteristic	ROC-AUC (95% CI)		
	SNOT-22 nasal domain scores	EPOS-defined nasal symptoms	*P* value
Lund-Mackay scores ≥5			
Nasal symptoms	0.59 (0.50-0.67)	0.53 (0.49-0.58)	.193
Nasal symptoms and reported duration of symptoms ≥3 months	0.61 (0.54-0.67)	0.53 (0.49-0.58)	.024
Lund-Mackay scores ≥1			
Nasal symptoms	0.59 (0.42-0.76)	0.58 (0.46-0.69)	.886
Nasal symptoms and reported duration of symptoms ≥3 months	0.61 (0.48-0.74)	0.58 (0.46-0.69)	.590

Abbreviations: EPOS, European Position Paper on Rhinosinusitis and Nasal Polyps; ROC-AUC, receiver operating characteristic area under the curve; SNOT-22, 22-item Sinonasal Outcome Test.

Within the univariate ordinal regression models, worse SNOT-22 nasal domain scores were associated with a significantly increased odds of a worse Lund-Mackay score (3.57; 95% CI, 1.89-6.74; *P* < .001; **Table 3**). Meeting the EPOS nasal symptom diagnostic criteria was not statistically significantly associated with CT results (odds ratio, 3.10; 95% CI, 1.27-7.59; *P* = .013; **Table 3**) when appropriately accounting for multiple comparisons. When both approaches to assessing symptoms were assessed simultaneously in the same regression analyses, the odds of an abnormal Lund-Mackay score were 3.11 (95% CI, 1.59-6.09; *P* = .001) times higher with worse SNOT-22 nasal scores. When EPOS nasal symptom criteria were met, the odds ratio was 1.87 (95% CI, 0.70-5.02; *P* = .213; **Table 3**).

**Table 3.** Ordinal Regression Models Assessing for Association Between SNOT-22 Nasal Domain Scores or EPOS-Defined Nasal Symptoms and Lund-Mackay Imaging Scores.

	Odds ratio (95% CI)	*P* value
Symptoms alone		
Univariate		
SNOT-22 nasal score	3.57 (1.89-6.74)	<.001
EPOS nasal symptoms	3.10 (1.27-7.59)	.013
Multivariate		
SNOT-22 nasal score	3.11 (1.59-6.09)	.001
EPOS nasal symptoms	1.87 (0.70-5.02)	.213
Symptoms and duration ≥3 months		
Univariate		
SNOT-22 Nasal Score	3.14 (1.58-6.23)	.001
EPOS Nasal Symptoms	3.95 (1.30-11.98)	.015
Multivariate		
SNOT-22 Nasal Score	2.82 (1.40-5.65)	.004
EPOS Nasal Symptoms	3.08 (0.97-9.76)	.056

Abbreviations: EPOS, European Position Paper on Rhinosinusitis and Nasal Polyps; SNOT-22, 22-item Sinonasal Outcome Test.

### Symptoms With a Reported Duration of at Least 3 Months

When a symptom duration of at least 3 months was also stipulated and Lund-Mackay scores ≥5 were required, the SNOT-22 nasal domain had seemingly better discriminatory capability than EPOS-defined nasal symptoms (SNOT-22 ROC-AUC, 0.61; EPOS ROC-AUC, 0.53). However, accounting for multiple comparisons, the related *P* value was nonsignificant, so there was no demonstrated difference among the 2 approaches (*P* = .0238). When a duration of at least 3 months of symptoms and a Lund-Mackay threshold of ≥1 was used (SNOT-22 ROC-AUC, 0.61; EPOS ROC-AUC, 0.58; *P* = .590; **Table 2**), overall, there was no difference in discriminatory capability between the SNOT-22 nasal domain and EPOS nasal symptoms.

When univariate ordinal regression was performed, the odds of an abnormal Lund-Mackay score were 3.14 (95% CI, 1.58-6.23) times higher with worse SNOT-22 nasal domain scores. These findings were statistically significant for the SNOT-22 nasal domain (*P* = .001) and not significant for the EPOS nasal symptom diagnostic criteria when accounting for multiple comparisons (odds ratio, 3.95; 95% CI, 1.30-11.98; *P* = .015). When assessed simultaneously through an ordinal regression analysis, worse SNOT-22 nasal scores were associated with a 2.82 (95% CI, 1.40-5.65) times higher odds of a worsened Lund-Mackay score (*P* = .004), while meeting the EPOS nasal symptom diagnostic criteria led to an odds ratio of 3.08 (95% CI, 0.970-9.76) for an abnormal Lund-Mackay score (*P* = .056); this latter finding was not statistically significant.

### SNOT-22 Overall Scores

The SNOT-22 overall score had low discriminatory capability of SNOT-22 for Lund-Mackay scores ≥5 (ROC-AUC, 0.53; 95% CI, 0.44-0.62) and Lund-Mackay scores ≥1 (ROC-AUC, 0.52; 95% CI, 0.34-0.71). In ordinal regression analyses, the SNOT-22 overall score did not significantly increase the odds of an abnormal Lund-Mackay score (odds ratio, 1.01; 95% CI, 0.99-1.03; *P* = .241).

## Discussion

These data suggested that the SNOT-22 nasal domain and EPOS-defined nasal symptoms had similar discriminatory capability for Lund-Mackay imaging scores, regardless of whether a 3-month duration of symptoms was also stipulated. Ordinal regression analyses, however, demonstrated that worse SNOT-22 nasal domain scores were associated with a significantly higher odds of abnormal Lund-Mackay scores. Satisfying the EPOS-defined nasal symptom diagnostic criteria also seemed to increase the odds of abnormal Lund-Mackay scores, although this was narrowly not statistically significant when accounting for multiple comparisons.

Prior studies of the discriminatory ability of patient reports have had mixed results. Some studies have demonstrated poor association with chronic rhinosinusitis, as related symptoms may be nonspecific.^3-6^ For example, authors have suggested that facial pain, dental pain, ear pain, and headache could be removed from diagnostic criteria without significant changes in diagnostic sensitivity.^5^ In contrast, other authors have concluded that questionnaire results can have functional sensitivity and specificity.^45^ Prior studies have also demonstrated that even within a single validated instrument, results may be mixed, with SNOT-22 nasal domain scores performing better than overall scores,^7-10,21-26,42^ also suggesting that specific approaches to symptoms and symptom clusters can alter discriminatory capacity. With regard to EPOS, 1 prior study investigated magnetic resonance imaging (MRI) and seasonality, while another assessed those who had imaging for nonrhinological reasons.^27,28^ We build upon this literature by conducting a direct comparison of the SNOT-22 and EPOS symptom criteria, focusing on CT scans in a broader-based population, and discretely incorporating the variable of symptom duration, which was not the focus of prior studies.

Amid the current coronavirus disease 2019 (COVID-19) practice environment,^46-53^ otolaryngologists have faced the challenge of making diagnoses without the typical reliance on concurrent fiberoptic exam. During telehealth encounters, we often need to proceed with medical decisions based on described symptoms and histories. With this in mind, we have experienced a renewed interest in the ability of isolated subjective symptom reports to predict objective findings such as imaging results. These symptoms are also often importantly used to determine whether a patient should be seen in person for further evaluation (eg, imaging) and/or surgical consideration, as well as to what degree and timing such follow-up should occur.^13^ It has thus become incumbent upon us to discern which symptom components and combinations are most useful when diagnosing conditions that would have readily relied on potentially aerosol-generating procedures such as nasal endoscopy for assessment in the past.

When taking a history, we have traditionally asked patients and families to report symptoms in a gestalt or dichotomous way (eg, How have your sinuses been? or Do you have facial pain?). This approach is often sufficient to determine whether patients meet the symptom component of diagnostic criteria such as those used in EPOS, which defines key symptoms according to their presence or absence, further qualified by a specified duration.^19,20^ Over time, incorporation of validated instruments such as SNOT-22 into daily practice has become more commonplace, such that patients may routinely receive more in-depth assessments of severity across a broader set of symptoms.^54,55^ These data suggest that SNOT-22 nasal domain scores and EPOS-defined symptoms have similar discriminatory capacity for Lund-Mackay results when assessed via ROC-AUC, while SNOT-22 nasal domain scores have a significant association with Lund-Mackay scores when assessed via ordinal regression models. These data suggest that SNOT-22 nasal domain scores could provide added diagnostic value. These results are particularly relevant in the current practice setting in which we first need to assess based on symptoms alone, as physical exam and imaging more frequently cannot be simultaneously obtained relative to concern for risks and/or patient deferral of in-person visits to health care facilities amid the current pandemic.^51-53^

Our study also assessed symptoms alone separately from symptoms with a specified duration of at least 3 months. The relationship between subjective symptoms and objective imaging findings was similar in both cases. In other cases, however, the serial addition of diagnostic characteristics may change the ultimate odds of disease.^56^ We assessed symptoms and duration in a stepwise fashion for this reason, as well as because the SNOT-22 instrument classically focuses on the preceding 4 weeks, rather than 3 months. Here, we found that the separately reported duration of sinonasal problems did not increase the successive likelihood of SNOT-22 nasal domain scores or EPOS-defined symptoms in predicting an abnormal Lund-Mackay imaging result within the current study’s context. Overall, the present data demonstrated that symptoms alone could not strongly predict CT findings of chronic rhinosinusitis, suggesting utility in corroboration of findings.

This study has limitations. These include taking place at a single academic institution and using just 2 different measures of patients’ symptoms, SNOT-22 and EPOS. Moreover, while our study has implications for telehealth delivery during the COVID-19 pandemic and beyond, it does not directly study the use of SNOT-22 and EPOS during telehealth visits. This may be a benefit as our “gold standard” is the diagnostic capability and timing we had prior to pandemic-related limitations. In addition, Lund-Mackay thresholds of import are not definitively established,^57,58^ and so we evaluated 2 proposed inflection points,^28^ which have limitations. As we continue to collect data, future studies could be conducted to assess the actual predictive value of SNOT-22 collected during a telehealth visit for CT scans in comparison to previous traditional visits. However, such a study could be limited by any extended duration between SNOT-22 completion and subsequent CT amid the pandemic, which could ultimately measure different actual severities of rhinosinusitis, which have changed over time.

## Conclusions

SNOT-22 and EPOS-defined nasal symptom assessments have similar discriminatory capability for Lund-Mackay scores when evaluated via ROC-AUC, regardless of symptom duration. Ordinal regression, however, demonstrated that the SNOT-22 nasal domain has a significant association with Lund-Mackay scores, more so than the EPOS-defined symptom set. As both SNOT-22 and EPOS symptom sets alone have limitations in discriminatory ability, further inquiries into optimizing patient-reported symptom content and patterns would provide additional value.

## References

[1] Centers for Disease Control and Prevention. Chronic sinusitis. Accessed May 11, 2020 https://www.cdc.gov/nchs/fastats/sinuses.htm

[2] Caulley L Thavorn K Rudmik L Cameron C Kilty SJ. Direct costs of adult chronic rhinosinusitis by using 4 methods of estimation: results of the US Medical Expenditure Panel Survey. J Allergy Clin Immunol. 2015;136(6):1517-1522. 26483176 10.1016/j.jaci.2015.08.037

[3] Bhattacharyya N. Clinical and symptom criteria for the accurate diagnosis of chronic rhinosinusitis. Laryngoscope. 2006;116(7, pt 2)(suppl 110):1-22. 10.1097/01.mlg.0000224508.59725.19 16826087

[4] Hsueh WD Conley DB Kim H, et al Identifying clinical symptoms for improving the symptomatic diagnosis of chronic rhinosinusitis. Int Forum Allergy Rhinol. 2013;3(4):307-314. 23129294 10.1002/alr.21106 PMC3622790

[5] Hirsch SD Reiter ER DiNardo LJ Wan W Schuman TA. Elimination of pain improves specificity of clinical diagnostic criteria for adult chronic rhinosinusitis. Laryngoscope. 2017;127(5):1011-1016. 28059446 10.1002/lary.26442

[6] Bhattacharyya N Lee LN. Evaluating the diagnosis of chronic rhinosinusitis based on clinical guidelines and endoscopy. Otolaryngol Head Neck Surg. 2010;143(1):147-151. 20620634 10.1016/j.otohns.2010.04.012

[7] Dejaco D Riedl D Huber A, et al The SNOT-22 factorial structure in European patients with chronic rhinosinusitis: new clinical insights. Eur Arch Otorhinolaryngol. 2019;276(5):1355-1365. 30739177 10.1007/s00405-019-05320-z PMC6458972

[8] Bhattacharyya T Piccirillo J Wippold FJ. Relationship between patient-based descriptions of sinusitis and paranasal sinus computed tomographic findings. Arch Otolaryngol Head Neck Surg. 1997;123(11):1189-1192. 9366698 10.1001/archotol.1997.01900110039006

[9] Sedaghat AR Gray ST Caradonna SD Caradonna DS. Clustering of chronic rhinosinusitis symptomatology reveals novel associations with objective clinical and demographic characteristics. Am J Rhinol Allergy. 2015;29(2):100-105. 25785749 10.2500/ajra.2015.29.4140

[10] Brooks SG Trope M Blasetti M, et al Preoperative Lund-Mackay computed tomography score is associated with preoperative symptom severity and predicts quality-of-life outcome trajectories after sinus surgery. Int Forum Allergy Rhinol. 2018;8(6):668-675. 29517156 10.1002/alr.22109 PMC5995576

[11] Tait S Kallogjeri D Suko J Kukuljan S Schneider J Piccirillo JF. Effect of budesonide added to large-volume, low-pressure saline sinus irrigation for chronic rhinosinusitis: a randomized clinical trial. JAMA Otolaryngol. Head Neck Surg. 2018;144(7):605-612. 29879268 10.1001/jamaoto.2018.0667 PMC6145785

[12] Amali A Saedi B Rahavi-Ezabadi S Ghazavi H Hassanpoor N. Long-term postoperative azithromycin in patients with chronic rhinosinusitis: a randomized clinical trial. Am J Rhinol Allergy. 2015;29(6):421-424. 26637580 10.2500/ajra.2015.29.4244

[13] Rudmik L Soler ZM Mace JC DeConde AS Schlosser RJ Smith TL. Using preoperative SNOT-22 score to inform patient decision for endoscopic sinus surgery. Laryngoscope. 2015;125(7):1517-1522. 25546168 10.1002/lary.25108 PMC4481170

[14] DeConde AS Mace JC Bodner T, et al SNOT-22 quality of life domains differentially predict treatment modality selection in chronic rhinosinusitis. Int Forum Allergy Rhinol. 2014;4(12):972-979. 25323055 10.1002/alr.21408 PMC4260999

[15] Soler ZM Rudmik L Hwang PH Mace JC Schlosser RJ Smith TL. Patient-centered decision making in the treatment of chronic rhinosinusitis. Laryngoscope. 2013;123(10):2341-2346. 23856802 10.1002/lary.24027 PMC3788096

[16] Hopkins C Rudmik L Lund VJ. The predictive value of the preoperative Sinonasal Outcome Test-22 score in patients undergoing endoscopic sinus surgery for chronic rhinosinusitis. Laryngoscope. 2015;125(8):1779-1784. 25891944 10.1002/lary.25318

[17] Caulley L Lasso A Rudmik L Kilty SJ. Pre-treatment SNOT-22 score predicts response to Endoscopic Polypectomy in Clinic (EPIC) our experience in 30 adults. Clin Otolaryngol. 2017;42(3):732-734. 26802485 10.1111/coa.12623

[18] DeConde AS Bodner TE Mace JC Smith TL. Response shift in quality of life after endoscopic sinus surgery for chronic rhinosinusitis. JAMA Otolaryngol Head Neck Surg. 2014;140(8):712-719. 25074504 10.1001/jamaoto.2014.1045 PMC4151456

[19] Fokkens WJ Lund VJ Mullol J, et al EPOS 2012: European position paper on rhinosinusitis and nasal polyps 2012: a summary for otorhinolaryngologists. Rhinology. 2012;50(1):1-12. 22469599 10.4193/Rhino12.000

[20] Fokkens WJ Lund VJ Hopkins C, et al European position paper on rhinosinusitis and nasal polyps 2020. Rhinology. 2020;58(suppl 29):1-464. 10.4193/Rhin20.600 32077450

[21] Bradley DT Kountakis SE. Correlation between computed tomography scores and symptomatic improvement after endoscopic sinus surgery. Laryngoscope. 2005;115(3):466-469. 15744159 10.1097/01.mlg.0000157840.55659.62

[22] Wabnitz DAM Nair S Wormald PJ Correlation between preoperative symptom scores, quality-of-life questionnaires, and staging with computed tomography in patients with chronic rhinosinusitis. Am J Rhinol. 2005;19(1):91-96. 15794082

[23] Ryan WR Ramachandra T Hwang PH. Correlations between symptoms, nasal endoscopy, and in-office computed tomography in post-surgical chronic rhinosinusitis patients. Laryngoscope. 2011;121(3):674-678. 21305550 10.1002/lary.21394

[24] Garneau J Ramirez M Armato SG, et al Computer-assisted staging of chronic rhinosinusitis correlates with symptoms. Int Forum Allergy Rhinol. 2015;5(7):637-642. 25854318 10.1002/alr.21499 PMC4509627

[25] Lim S Ramirez MV Garneau JC, et al Three-dimensional image analysis for staging chronic rhinosinusitis. Int Forum Allergy Rhinol. 2017;7(11):1052-1057. 28941169 10.1002/alr.22014 PMC5965263

[26] Zheng Y Zhao Y Lv D, et al Correlation between computed tomography staging and quality of life instruments in patients with chronic rhinosinusitis. Am J Rhinol Allergy. 2010;24(1):e41-e45. 20109323 10.2500/ajra.2010.24.3430

[27] Dietz de Loos D Lourijsen ES Wildeman MAM, et al Prevalence of chronic rhinosinusitis in the general population based on sinus radiology and symptomatology. J Allergy Clin Immunol. 2019;143(3):1207-1214. 30578880 10.1016/j.jaci.2018.12.986

[28] del Rio A Trost N Tartaglia C O’Leary SJ Michael P. Seasonality and incidental sinus abnormality reporting on MRI in an Australian climate. Rhinology. 2012;50(3):319-324. 22888491 10.4193/Rhino11.270

[29] Zhao L Yu KN Tan JL, et al Severity of rhinosinusitis: comparison between visual analog scale given by patients and otorhinolaryngologists [published online 5 13, 2020]. Am J Rhinol Allergy. 10.1177/1945892420923934 32403940

[30] Morley AD Sharp HR. A review of sinonasal outcome scoring systems—which is best? Clin Otolaryngol. 2006;31(2):103-109. 16620328 10.1111/j.1749-4486.2006.01155.x

[31] Hopkins C Gillett S Slack R Lund VJ Browne JP. Psychometric validity of the 22-item Sinonasal Outcome Test. Clin Otolaryngol. 2009;34(5):447-454. 19793277 10.1111/j.1749-4486.2009.01995.x

[32] Quintanilla-Dieck L Litvack JR Mace JC Smith TL. Comparison of disease-specific quality-of-life instruments in the assessment of chronic rhinosinusitis. Int Forum Allergy Rhinol. 2012;2(6):437-443. 22696495 10.1002/alr.21057 PMC3443528

[33] Feng AL Wesely NC Hoehle LP, et al A validated model for the 22-item Sino-Nasal Outcome Test subdomain structure in chronic rhinosinusitis. Int Forum Allergy Rhinol. 2017;7(12):1140-1148. 29028287 10.1002/alr.22025

[34] Lund VJ Mackay IS. Staging in rhinosinusitus. Rhinology. 1993;31(4):183-184. 8140385

[35] Oluwole M Russell N L. Tan Gardiner Q White P. A comparison of computerized tomographic staging systems in chronic sinusitis. Clin Otolaryngol Allied Sci. 1996;21(1):91-95. 8674232

[36] Metson R Gliklich RE Stankiewicz JA, et al Comparison of sinus computed tomography staging systems. Otolaryngol Head Neck Surg. 1997;117(4):372-379. 9339799 10.1016/S0194-5998(97)70129-3

[37] Ashraf N Bhattacharyya N. Determination of the “incidental” Lund score for the staging of chronic rhinosinusitis. Otolaryngol Head Neck Surg. 2001;125(5):483-486. 11700446 10.1067/mhn.2001.119324

[38] Akobeng AK. Understanding diagnostic tests 3: receiver operating characteristic curves. Acta Paediatr. 2007;96(5):644-647. 17376185 10.1111/j.1651-2227.2006.00178.x

[39] DeLong ER DeLong DM Clarke-Pearson DL. Comparing the areas under two or more correlated receiver operating characteristic curves: a nonparametric approach. Biometrics. 1988;44(3):837-845. 3203132

[40] Toma S Hopkins C. Stratification of SNOT-22 scores into mild, moderate or severe and relationship with other subjective instruments. Rhinology. 2016;54(2):129-133. 27017484 10.4193/Rhino15.072

[41] Chowdhury NI Mace JC Bodner TE, et al Investigating the minimal clinically important difference for SNOT-22 symptom domains in surgically managed chronic rhinosinusitis. Int Forum Allergy Rhinol. 2017;7(12):1149-1155. 29053911 10.1002/alr.22028 PMC5716928

[42] Zhou AS Prince AA Maxfield AZ Shin JJ. Psychological status as an effect modifier of the association between sinonasal instrument and imaging results [published online 5 26, 2020]. Otolaryngol Head Neck Surg. 10.1177/0194599820926129 32450735

[43] McKelvey RD Zavoina W. A statistical model for the analysis of ordinal level dependent variables. J Math Sociol. 1975;4(1):103-120.

[44] Benjamini Y Hochberg Y. Controlling the false discovery rate: a practical and powerful approach to multiple testing. J R Stat Soc Ser B. 1995;57(1):289-300.

[45] Workman AD Parasher AK Blasetti MT Palmer JN Adappa ND Glicksman JT. Accuracy of self-reported diagnosis of chronic rhinosinusitis. Otolaryngol Head Neck Surg. 2019;160(3):556-558. 30396322 10.1177/0194599818811328

[46] Zhao C Viana A Wang Y Wei H-Q Yan A-H Capasso R. Otolaryngology during COVID-19: preventive care and precautionary measures. Am J Otolaryngol. 2020;41(4):102508. 32345446 10.1016/j.amjoto.2020.102508 PMC7195080

[47] Ralli M Greco A de Vincentiis M. The effects of the COVID-19/SARS-CoV-2 pandemic outbreak on otolaryngology activity in Italy [published online 4 29, 2020]. Ear Nose Throat J. 10.1177/0145561320923893 32347112

[48] Civantos FJ Leibowitz JM Arnold DJ, et al Ethical surgical triage of patients with head and neck cancer during the COVID-19 pandemic. Head Neck. 2020;42(7):1423-1447. 32357378 10.1002/hed.26229 PMC7267510

[49] Brody RM Albergotti WG Shimunov D Nicolli E Harris BN Bur AM. Changes in head and neck oncologic practice during the COVID-19 pandemic. Head Neck. 2020;42(7):1448-1453. 32357380 10.1002/hed.26233 PMC7267666

[50] Tay JK Lim WS Loh WS Loh KS. Sustaining otolaryngology services for the long haul during the COVID-19 pandemic: experience from a tertiary health system. Otolaryngol Head Neck Surg. 2020;163(1):47-50. 32366174 10.1177/0194599820922983

[51] Cheng X Liu J Li N, et al Otolaryngology providers must be alert for patients with mild and asymptomatic COVID-19. Otolaryngol Head Neck Surg. 2020;162(6):809-810. 32286913 10.1177/0194599820920649

[52] Workman AD Welling DB Carter BS, et al Endonasal instrumentation and aerosolization risk in the era of COVID-19: simulation, literature review, and proposed mitigation strategies. Int Forum Allergy Rhinol. 2020;10(7):798-805. 32243678 10.1002/alr.22577

[53] Patel ZM Fernandez-Miranda J Hwang PH, et al Letter: precautions for endoscopic transnasal skull base surgery during the COVID-19 pandemic. Neurosurgery. 2020;87(1):E66-E67. 32293678 10.1093/neuros/nyaa125 PMC7184431

[54] Shin JJ Carroll TL Prince AA Landman AB. The utility and feasibility of extending beyond traditional patient descriptions in daily practice. Laryngoscope. 2020;130(suppl 3):S1-S13. 10.1002/lary.28467 31876293

[55] Shin JJ. An electronic interface to routinize outcomes assessment and streamline clinic workflow. Laryngoscope. 2017;127(5):1058-1060. 27377677 10.1002/lary.26160

[56] Shin JJ Stinnett SS Randolph GW. Evidence-based medicine in otolaryngology part 4: everyday probabilities—nonbinary diagnostic tests. Otolaryngol Head Neck Surg. 2013;149(2):179-186. 23715687 10.1177/0194599813491070

[57] Bhattacharyya N Fried MP. The accuracy of computed tomography in the diagnosis of chronic rhinosinusitis. Laryngoscope. 2003;113(1):125-129. 12514395 10.1097/00005537-200301000-00023

[58] Hopkins C Browne JP Slack R Lund V Brown P. The Lund-Mackay staging system for chronic rhinosinusitis: how is it used and what does it predict? Otolaryngol Head Neck Surg. 2007;137(4):555-561. 17903570 10.1016/j.otohns.2007.02.004

